# The Current State of Robot-Assisted Minimally Invasive Esophagectomy (RAMIE): Outcomes from the Upper GI International Robotic Association (UGIRA) Esophageal Registry

**DOI:** 10.1245/s10434-024-16364-9

**Published:** 2024-11-04

**Authors:** Cezanne D. Kooij, Cas de Jongh, B. Feike Kingma, Mark I. van Berge Henegouwen, Suzanne S. Gisbertz, Yin-Kai Chao, Philip W. Chiu, Philippe Rouanet, Anne Mourregot, Arul Immanuel, Tom Mala, Gijs I. van Boxel, Nicholas C. Carter, Hecheng Li, Hans F. Fuchs, Christiane J. Bruns, Simone Giacopuzzi, Jörg C. Kalff, Jens-Peter Hölzen, Mazen A. Juratli, Frank Benedix, Eric Lorenz, Jan-Hendrik Egberts, Jan W. Haveman, Boudewijn van Etten, Beat P. Müller, Peter P. Grimminger, Felix Berlth, Guillaume Piessen, Jan W. van den Berg, Marco Milone, James D. Luketich, Inderpal S. Sarkaria, Rubens A. A. Sallum, Marc J. van Det, Ewout A. Kouwenhoven, Matthias Brüwer, Tomas Harustiak, Takahiro Kinoshita, Takeo Fujita, Hiroyuki Daiko, Zhigang Li, Jelle P. Ruurda, Richard van Hillegersberg, UGIRA Study Group

**Affiliations:** 1 University Medical Center Utrecht, University Utrecht, Utrecht, The Netherlands; 2 Amsterdam UMC Location University of Amsterdam, Amsterdam, The Netherlands; 3 Cancer Center Amsterdam, Amsterdam, The Netherlands; 4 Chang Gung Memorial Hospital-Linko, Chang Gung University, Taoyuan, Taiwan; 5 Faculty of Medicine, The Chinese University of Hong Kong, Hong Kong SAR, China; 6 Montpellier Cancer Institute, Montpellier, France; 7 Royal Victoria Infirmary Newcastle Upon Tyne, Newcastle upon Tyne, UK; 8 Department of Gastrointestinal Surgery, Oslo University Hospital and Institute of Clinical Medicine, University of Oslo, Oslo, Norway; 9 Portsmouth Hospitals NHS Trust, Portsmouth, UK; 10 Ruijin Hospital, Shanghai Jiao Tong University School of Medicine, Shanghai, China; 11 University of Cologne, Cologne, Germany; 12 University of Verona, Verona, Italy; 13 University Hospital of Bonn, Bonn, Germany; 14 Universitätsklinikum Münster, Münster, Germany; 15 University Hospital Magdeburg, Magdeburg, Germany; 16 Israelitisches Krankenhaus Hamburg, Hamburg, Germany; 17 University Medical Center Groningen, University of Groningen, Groningen, The Netherlands; 18 University Digestive Healthcare, Basel, Switzerland; 19 University Medical Center of the Johannes Gutenberg University, Mainz, Germany; 20 Univ. Lille, CNRS, Inserm, CHU Lille, UMR9020-U1277 – CANTHER – Cancer Heterogeneity Plasticity and Resistance to Therapies, Lille, France; 21 “Federico II” University of Naples, Naples, Italy; 22 University Pittsburgh Medical Center, Pittsburgh, PA USA; 23 University of Texas Southwestern Medical Center, Dallas, TX USA; 24 University of São Paulo, São Paulo, Brazil; 25 ZGT Almelo, Almelo, The Netherlands; 26 St. Franziskus Hospital, Münster, Germany; 27 Motol University Hospital, First Faculty of Medicine, Charles University, Prague, Czech Republic; 28 National Cancer Center Hospital East, Chiba, Japan; 29 National Cancer Center Hospital, Tokyo, Japan; 30 Shanghai Chest Hospital, Shanghai Jiao Tong University School of Medicine, Shanghai, China

**Keywords:** Esophageal cancer, Robot-assisted esophagectomy, Minimally invasive esophagectomy

## Abstract

**Background:**

Robot-assisted minimally invasive esophagectomy (RAMIE) is increasingly adopted in centers worldwide, with ongoing refinements to enhance results. This study aims to assess the current state of RAMIE worldwide and to identify potential areas for improvement.

**Methods:**

This descriptive study analyzed prospective data from esophageal cancer patients who underwent transthoracic RAMIE in Upper GI International Robotic Association (UGIRA) centers. Main endpoints included textbook outcome rate, surgical techniques, and perioperative outcomes. Analyses were performed separately for intrathoracic (Ivor–Lewis) and cervical anastomosis (McKeown), divided into three time cohorts (2016–2018, 2019–2020, 2021–2023). A sensitivity analysis was conducted with cases after the learning curve (> 70 cases).

**Results:**

Across 28 UGIRA centers, 2012 Ivor–Lewis and 1180 McKeown procedures were performed. Over the time cohorts, textbook outcome rates were 39%, 48%, and 49% for Ivor–Lewis, and 49%, 63%, and 61% for McKeown procedures, respectively. Fully robotic procedures accounted for 66%, 51%, and 60% of Ivor–Lewis procedures, and 53%, 81%, and 66% of McKeown procedures. Lymph node yield showed 27, 30, and 30 nodes in Ivor–Lewis procedures, and 26, 26, and 34 nodes in McKeown procedures. Furthermore, high mediastinal lymphadenectomy was performed in 65%, 43%, and 37%, and 70%, 48%, and 64% of Ivor–Lewis and McKeown procedures, respectively. Anastomotic leakage rates were 22%, 22%, and 16% in Ivor–Lewis cases, and 14%, 12%, and 11% in McKeown cases. Hospital stay was 13, 14, and 13 days for Ivor–Lewis procedures, and 12, 9, and 11 days for McKeown procedures. In Ivor–Lewis and McKeown, respectively, the sensitivity analysis revealed textbook outcome rates of 43%, 54%, and 51%, and 47%, 64%, and 64%; anastomotic leakage rates of 28%, 18%, and 15%, and 13%, 11%, and 10%; and hospital stay of 11, 12, and 12 days, and 10, 9, and 9 days.

**Conclusions:**

This study demonstrates favorable outcomes over time in achieving textbook outcome after RAMIE. Areas for improvement include a reduction of anastomotic leakage and shortening of hospital stay.

**Supplementary Information:**

The online version contains supplementary material available at 10.1245/s10434-024-16364-9.

Esophageal cancer stands as a global health challenge, ranking as the eighth most commonly diagnosed cancer and the sixth leading cause of cancer-related mortality worldwide, with projections indicating a concerning increase due to population growth and aging.^1,2^ However, the burden of esophageal cancer reveals heterogeneity across nations and demographic groups, which can be attributed to risk factors and distribution of subtypes, notably adenocarcinoma and squamous cell carcinoma.^3,4^ The current standard of care for locally advanced disease involves a multimodal approach, with radical esophagectomy as the cornerstone of treatment, achieving up to 50% 5-year survival rates.^5–7^

The surgical approach for esophageal cancer has evolved over the past decades. Traditionally, esophageal cancer surgery was performed via an open approach, which is associated with high postoperative morbidity and mortality.^7^ Minimally invasive esophagectomy (MIE) was introduced in clinical practice aiming to reduce surgical trauma by avoiding thoracotomy and/or laparotomy and therefore reducing postoperative morbidity and enhancing recovery. The TIME trial, a randomized controlled study comparing open esophagectomy with MIE, demonstrated short-term benefits of MIE by reducing cardiorespiratory complications.^8^ Additionally, over the last two decades, the introduction of robot-assisted MIE (RAMIE) was demonstrated to be safe, feasible, and superior to open esophagectomy in terms of postoperative complications, pain, and short-term quality of life with similar long-term survival.^9,10^ However, potential superiority of RAMIE over MIE is yet to be established, and definitive conclusions await the (long-term) results of ongoing and recently finished clinical trials (REVATE trial and ROBOT-2 trial).^11,12^ Additionally, it is worth noting that RAMIE is a complex procedure associated with a significant learning curve, as indicated in the literature, with case numbers ranging from 20 to 80, depending on the studied outcome and surgical/robotic experience.^13^

Previously, the Upper GI International Robotic Association (UGIRA) study group provided a global overview of RAMIE techniques based on the first results from the UGIRA Esophageal Registry, a multicenter, international collaboration.^14^ With a growing number of centers adopting RAMIE in clinical practice, and ongoing refinements and adjustments in techniques to enhance oncological and postoperative results, this study aimed to assess the current state of RAMIE within this worldwide cohort, to evaluate outcomes over time, and to identify potential areas for improvement.

## Methods

In 2017, the UGIRA was established by a multicontinental group of surgeons to facilitate the implementation and advancement of robotic esophagogastric surgery. The UGIRA Esophageal Registry, also initiated in 2017, aimed to gain insight into the worldwide techniques and outcomes of robotic esophageal procedures.^14^ Since the start of this registry, an increasing number of centers worldwide have joined the UGIRA, resulting in a continuous expansion of the prospective data collection in this registry. Central ethics approval was obtained from the University Medical Center Utrecht (17/837), waiving informed consent as no personal data were collected. Institutional Review Board approval was acquired in each participating center. The UGIRA Scientific Committee reviewed and approved this research, and the paper adheres to the Strengthening the Reporting of Observational Studies (STROBE) guidelines for observational studies.^15^

### Patient Population and Study Design

This observational cohort study utilizes the prospectively collected data from the UGIRA Esophageal Registry, and includes data from esophageal cancer patients (cT1-4N0-3M0) who underwent transthoracic RAMIE with curative intent between 2016 and December 2023. Centers that performed and registered at least 20 RAMIE cases were eligible for this study. Exclusion criteria encompassed histology other than adenocarcinoma or squamous cell carcinoma, incomplete data regarding the year of procedure, and/or missing data for >25% of variables.

### Outcomes

The primary endpoint was textbook outcome rate, previously defined as a composite measure including tumor-negative resection margins, ≥ 15 lymph nodes at pathology, no intraoperative complications, no Clavien–Dindo ≥ 3 complications, no intensive or medium care unit (ICU/MCU) readmission, no hospital stay > 21 days, no re-interventions, no hospital readmission after discharge, and no postoperative mortality.^16^ Secondary endpoints included surgical techniques, encompassing use of the robot, patient positioning, operative time, anastomotic technique and type, and (extent of) lymphadenectomy, perioperative complications, radical resection (R0) rates as defined by the College of American Pathologists, lymph node yield, length of hospital stay and ICU/MCU stay, and 30-day mortality.

### Statistical Analysis

All analyses were performed using IBM SPSS Statistics 27.0.1.0 (IBM Corporation, Armonk, NY, USA). Analyses were performed separately for patients undergoing RAMIE with intrathoracic (Ivor–Lewis) or cervical (McKeown) anastomosis. The outcomes were descriptively analyzed for three time cohorts: 2016–2018, 2019–2020, and 2021–2023. Continuous data are depicted as means with standard deviations (SDs), or medians with range or interquartile range (IQR), depending on data distribution. Categorical data are shown as frequencies with percentages (%).

In a sensitivity analysis, based on a previous learning curve study, the initial 70 cases per center were excluded in order to minimize potential bias due to learning curve effects and case selection.^17^ This approach aimed to offer clearer insights into outcomes from experienced centers and to help identify potential areas for improvement.

## Results

### Patient Population

**Fig. 1:**
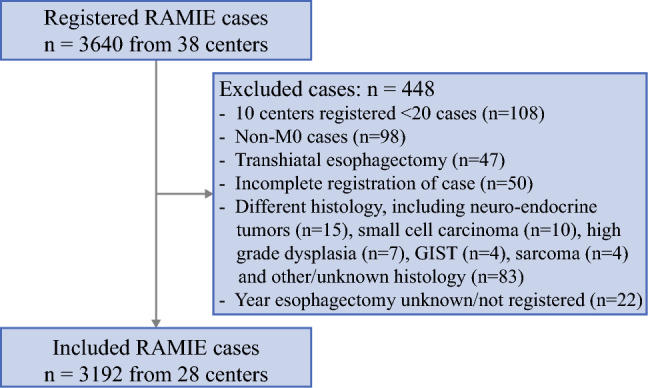
Inclusion of cases into the UGIRA Esophageal Registry. Date of extraction from online data capturing tool: 31 December 2023. *UGIRA* Upper GI International Robotic Association, *RAMIE* robot-assisted minimally invasive esophagectomy, *GIST* gastrointestinal stromal tumor

During the study period, 3640 RAMIE cases were registered in the UGIRA Esophageal Registry. After excluding 448 cases (Fig. 1), 3192 RAMIE cases from 28 UGIRA centers in Europe, Asia, North-America and South-America were included, showing 2012 Ivor–Lewis and 1180 McKeown RAMIE procedures.

The baseline characteristics are summarized in Table 1. In the Ivor–Lewis group, the median age was 66 years (IQR 59–71) and the majority were male (*n* = 1648, 82%), with almost all patients having an American Society of Anesthesiologists (ASA) score of ≥ 2 (*n* = 1840, 91%). Most of these patients (*n* = 1494, 74%) had at least one comorbidity, mostly cardiac (*n* = 633, 32%) or vascular (*n* = 549, 27%). Adenocarcinoma was most common (*n* = 1683, 84%) and most patients were diagnosed with stage cT3 disease (*n* = 1373, 68%), with clinically suspected lymph node metastases in 64% (*n* = 1295) of cases. Neoadjuvant treatment consisted of chemoradiotherapy (*n* = 1067, 53%), chemotherapy (*n* = 626, 31%), or upfront surgery without neoadjuvant therapy (*n* = 290, 15%).

**Table 1:** Baseline characteristics of the whole cohort (*n* = 3192) from 28 centers included in the UGIRA Esophageal Registry

	Ivor-Lewis [*n* = 2012] 24 centers	McKeown [*n* = 1180] 21 centers
Continent		
Europe	1872 (93)	245 (21)
North America	88 (5)	3 (0)
Asia	52 (3)	908 (77)
South America	0 (0)	24 (2)
Age, years (median [IQR])	66 [59–72]	66 [59–71]
Sex		
Male	1648 (82)	947 (80)
Female	363 (18)	233 (20)
Missing	1 (0)	0 (0)
BMI, kg/m^2^ (mean ± SD)	26.3 ± 4.7	23.4 ± 3.9
ASA classification		
1	136 (7)	55 (5)
2	1046 (52)	759 (64)
3	763 (38)	350 (30)
4	31 (2)	8 (1)
Missing	49 (2)	8 (1)
Any comorbidity	1494 (74)	607 (51)
Pulmonary comorbidity	299 (15)	82 (7)
Cardial comorbidity	633 (32)	133 (11)
Vascular comorbidity	549 (27)	277 (24)
Oncological comorbidity	184 (9)	76 (6)
Neurological comorbidity	114 (6)	31 (3)
Diabetes	304 (15)	134 (11)
Histology		
Adenocarcinoma	1683 (84)	169 (14)
Squamous cell carcinoma	329 (16)	1011 (86)
Clinical T stage		
cT1a	45 (2)	29 (3)
cT1b	118 (6)	80 (7)
cT2	372 (19)	330 (28)
cT3	1373 (68)	681 (58)
cT4a	66 (3)	24 (2)
cT4b	7 (0)	30 (3)
Missing	31 (2)	6 (1)
Clinical N stage		
cN0	699 (35)	334 (28)
cN+ (cN1–cN3)	1295 (64)	842 (71)
Missing	18 (1)	4 (0)
Neoadjuvant therapy		
None	290 (14)	490 (42)
Chemotherapy	626 (31)	239 (20)
Radiotherapy	4 (0)	5 (0)
Chemoradiotherapy	1067 (53)	413 (35)
Other	20 (1)	33 (3)
Missing	5 (0)	0 (0)
Robotic system		
Da Vinci S	2 (0)	227 (19)
Da Vinci Si	236 (12)	332 (28)
Da Vinci X	90 (5)	5 (0)
Da Vinci Xi	1684 (84)	616 (52)

Data are expressed as *n* (%) unless otherwise

Percentages may not equal 100% due to rounding

*BMI* body mass index, *ASA* American Society of Anesthesiologists, *SD* standard deviation, *IQR* interquartile range, *UGIRA* Upper GI International Robotic Association

In the McKeown patient group, median age was 66 years (IQR 59–71), 80% (*n* = 947) of patients were male, and 95% (*n* = 1117) of patients had an ASA score of ≥ 2. Half of the patients (*n* = 607, 51%) had at least one comorbidity, predominantly vascular comorbidities (*n* = 277, 24%). For McKeown, 42% (*n* = 490) did not undergo neoadjuvant treatment, with 79% (*n* = 386) originating from a single Asian center, while 35% (*n* = 413) received neoadjuvant chemoradiotherapy, and 20% (*n* = 239) received neoadjuvant chemotherapy.

### Primary Outcome

**Fig. 2:**
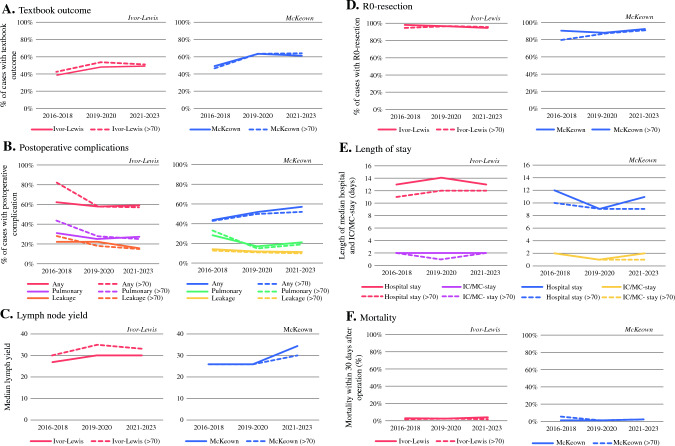
(**A**) Textbook outcome, (**B**) postoperative complications (specified for pulmonary complication and anastomotic leakage), (**C**) median lymph node yield, (**D**) R0 resection, (**E**) length of hospital and IC/MC stay, and (**F**) mortality within 30 days per time period for Ivor–Lewis (left) and McKeown (right) procedures in the whole cohort (solid line) and in the sensitivity analysis (dotted line), excluding the first 70 cases per center. Data from the UGIRA Esophageal Registry. *IC* intensive care, *MC* medium care, *UGIRA* Upper GI International Robotic Association

The Ivor–Lewis and McKeown cohorts were stratified based on three time periods (2016–2018, 2019–2020, and 2021–2023), resulting in three groups of 368, 563, and 1081 cases (Ivor–Lewis) and 275, 406, and 499 cases (McKeown), respectively. The number of centers contributing to each time period is depicted in electronic supplementary material (ESM) Table 1 and ESM Table 2 for Ivor–Lewis and McKeown cases, respectively. Over time, textbook outcome rates were 39%, 48%, and 49% for Ivor–Lewis procedures, and 49%, 63%, and 61% for McKeown procedures (Fig. 2a).

**Table 2:** Outcomes over time in surgical techniques in the UGIRA Esophageal Registry cohort (*n* = 3192), stratified based on year of operation

	Ivor–Lewis [*n* =2012] 24 centers			McKeown [*n* = 1180] 21 centers		
	2016–2018 [*n* = 368] 12 centers	2019–2020 [*n* = 563] 16 centers	2021–2023 [*n* = 1081] 20 centers	2016–2018 [*n* = 275] 11 centers	2019–2020 [*n* = 406] 15 centers	2021–2023 [*n* = 499] 17 centers
Use of robot						
Robot thorax + robot abdomen	241 (66)	287 (51)	652 (60)	146 (53)	328 (81)	327 (66)
Robot thorax + laparoscopy	108 (29)	212 (38)	332 (31)	119 (43)	64 (16)	141 (28)
Robot thorax + laparotomy	4 (1)	8 (1)	7 (1)	10 (4)	13 (3)	27 (5)
Robot abdomen + thoracoscopy	4 (1)	21 (4)	9 (1)	0 (0)	0 (0)	1 (0)
Robot abdomen + thoracotomy	11 (3)	35 (6)	81 (8)	0 (0)	1 (0)	3 (0)
Patient positioning, thoracic phase						
Semi-prone	250 (68)	421 (75)	737 (68)	158 (58)	98 (24)	124 (25)
Left lateral decubitus	118 (32)	117 (21)	273 (25)	117 (43)	294 (72)	253 (51)
Prone	0 (0)	15 (3)	71 (7)	0 (0)	14 (3)	116 (23)
Other	0 (0)	10 (2)	0 (0)	0 (0)	0 (0)	0 (0)
Lymphadenectomy						
Mediastinal lymphadenectomy						
High-paratracheal nodes	240 (65)	242 (43)	401 (37)	193 (70)	196 (48)	319 (64)
Mid-subcarinal nodes	353 (96)	417 (74)	879 (81)	265 (96)	376 (93)	461 (92)
Low-para-esophageal nodes	357 (97)	528 (94)	1034 (96)	211 (77)	330 (81)	471 (94)
Total lymph node yield (median [IQR])	27 [20–35]	30 [21–39]	30 [23–39]	26 [19–33]	26 [20–34]	34 [25–48]
Anastomosis						
Anastomotic technique						
Circular-stapled	163 (44)	369 (66)	689 (64)	129 (47)	289 (71)	274 (55)
Hand-sewn	171 (47)	80 (14)	135 (12)	85 (31)	42 (10)	77 (15)
Linear-stapled	34 (9)	114 (20)	257 (24)	47 (17)	75 (19)	147 (30)
No anastomosis	0 (0)	0 (0)	0 (0)	14 (5)	0 (0)	1 (0)
Anastomotic type						
End-to-side	243 (66)	452 (80)	748 (69)	195 (75)	323 (80)	310 (62)
End-to-end	88 (24)	6 (1)	67 (6)	17 (7)	22 (5)	144 (29)
Side-to-side	37 (10)	105 (19)	266 (25)	49 (19)	61 (15)	44 (9)
Postoperative outcomes						
Textbook outcome^a^	145 (39)	269 (48)	525 (49)	135 (49)	255 (63)	302 (61)
Operative time, min (median [IQR])	400 [360–465]	430 [374–497]	425 [368–490]	363 [245–462]	289 [245–370]	382 [284–465]
Postoperative complications						
Any	227 (62)	328 (58)	633 (59)	121 (44)	210 (52)	286 (57)
Pulmonary	114 (31)	141 (25)	288 (27)	77 (28)	69 (17)	205 (21)
Pneumonia	66 (18)	94 (17)	204 (19)	42 (15)	40 (10)	58 (12)
Anastomotic leakage	81 (22)	121 (22)	173 (16)	37 (14)	47 (12)	53 (11)
Recurrent laryngeal nerve injury	2 (1)	11 (2)	18 (2)	22 (8)	102 (25)	119 (24)
R0 resection^b^	354 (97)	538 (96)	999 (94)	247 (90)	357 (88)	451 (92)
Length of stay						
ICU/MCU stay, days (median [IQR])	2 [1–6]	2 [1–5]	2 [1–5]	2 [1–4]	1 [1–3]	2 [1–3]
Hospital stay, days (median [IQR])	13 [9–18]	14 [11–22]	13 [9–20]	12 [9–17]	9 [8–13]	11 [8–19]
Mortality < 30 days	10 (3)	17 (3)	38 (4)	5 (1)	5 (1)	10 (2)

Data are expressed as *n* (%) unless otherwise specified

Percentages may not equal 100% due to rounding

*IQR* interquartile range, *UGIRA* Upper GI International Robotic Association, *ICU* intensive care unit, *MCU* medium care unit

^a^Definition: complete resection (R0), no intraoperative complications, a lymph node yield of ≥15, no Clavien–Dindo ≥3 complications, no re-interventions, no readmission to the ICU/MCU, no hospital readmission after discharge, no length of hospital stay >21 days, no mortality <30 days, and no in-hospital mortality [16]

^b^Refers to R0 as defined by the College of American Pathologists (i.e. absence of malignant cells within the resection margins)

### Surgical Techniques and Intraoperative Results

Details regarding surgical techniques and postoperative outcomes over time are presented in Table 2.

In the Ivor–Lewis cohort, a fully robotic approach was adopted in 66%, 51%, and 60% of procedures. Patient positioning was mostly a semi-prone (68%, 75%, 68%) or left lateral decubitus (32%, 21%, 25%) position, with few patients in the prone position (0%, 3%, 7%).

A more extensive high mediastinal lymphadenectomy was conducted in 65%, 43%, and 37% of cases, while a mid or low mediastinal lymphadenectomy was performed in 96%, 74%, and 81%, and 97%, 94%, and 96% of cases, respectively. Median lymph node yield was 27, 30, and 30 nodes (Fig. 2c).

Circular stapling was used for the esophagogastric anastomosis in 44%, 66%, and 64% of procedures, linear stapling was used in 9%, 20%, and 24% of procedures, and a hand-sewn technique was used in 47%, 14%, and 12% of procedures.

The median duration of Ivor–Lewis procedures was 400, 430, and 425 min.

In the McKeown cohort, 53%, 81%, and 66% of procedures were fully robotically performed. Patients were either positioned in a semi-prone (58%, 24%, 25%), left lateral decubitus (43%, 72%, 51%), or prone position (0%, 3%, 23%).

A high mediastinal lymphadenectomy was performed in 70%, 48%, and 64% of cases, while a mid or low mediastinal lymphadenectomy was performed in 96%, 93%, and 92%, and 77%, 81%, and 94% of cases, respectively. Median lymph node yield was 26, 26, and 34 nodes (Fig. 2c).

Circular stapling was used for the esophagogastric anastomosis in 47%, 71%, and 55% of procedures, linear stapling was used in 17%, 19%, and 30% of procedures, and a hand-sewn technique was used in 31%, 10%, and 15% of procedures.

McKeown procedures had a median operative time of 363, 289, and 382 min.

### Postoperative Outcomes

In the Ivor–Lewis cohort, postoperative complication rates were 62%, 58%, and 59%. Pulmonary complications occurred in 31%, 25%, and 27% of patients, and anastomotic leakage occurred in 22%, 22%, and 16% of patients (Fig. 2b). Recurrent laryngeal nerve injury was reported in 1%, 2%, and 2% of patients.

R0 resection was confirmed in 97%, 96%, and 94% of cases (Fig. 2d). Median hospital stay was 13, 14, and 13 days, and ICU stay was 2, 2, and 2 days (Fig. 2e). Mortality within 30 days after surgery was 3%, 3%, and 4% (Fig. 2f).

In the McKeown cohort, postoperative complications were observed in 44%, 52%, and 57% of patients, with pulmonary complications occurring in 31%, 25%, and 27% of cases, and anastomotic leakage occurring in 14%, 12%, and 11% of cases (Fig. 2b). Recurrent laryngeal nerve injury was reported in 8%, 25%, and 24% of cases.

R0 resection was confirmed in 90%, 88%, and 92% of cases (Fig. 2d). Median hospital stay and ICU stay were 12, 9, and 11 days, and 2, 1, and 1 days, respectively (Fig. 2e). Mortality within 30 days after surgery was 1%, 1%, and 2% (Fig. 2f).

### Sensitivity Analysis

In the sensitivity analysis, the first 70 cases of each center were excluded, yielding 1609 cases from 14 experienced centers, of which 864 were Ivor–Lewis procedures and 745 were McKeown procedures. The baseline characteristics are comparable with the whole cohort (ESM Table 3). Stratification based on time period resulted in 47, 177, and 640 Ivor–Lewis cases, and 60, 351, and 334 McKeown cases. Textbook outcome rates were 43%, 54%, and 51% in Ivor–Lewis, and 47%, 64%, and 64% in McKeown procedures (Fig. 2a).

Table 3 shows the intraoperative and postoperative details, separately for Ivor–Lewis and McKeown procedures. A fully robotic approach was performed in 34%, 75%, and 54% of Ivor–Lewis cases, and 58%, 84%, and 83% of McKeown cases. In 80%, 67%, and 39% of Ivor–Lewis procedures, and 63%, 43%, and 49% of McKeown procedures, a high mediastinal lymphadenectomy was performed. Total median lymph node yield was 30, 35, and 33 nodes in Ivor–Lewis procedures, and 26, 26, and 30 nodes in McKeown procedures (Fig. 2c).

**Table 3:** Outcomes over time in surgical techniques in experienced centers (case 71 and onwards), including patients in the UGIRA Esophageal Registry, stratified based on year of operation

	Ivor–Lewis [*n* = 864] 11 centers			McKeown [*n* = 745] 12 centers		
	2016–2018 [*n* = 47] 3 centers	2019–2020 [*n* = 177] 5 centers	2021–2023 [*n* = 640] 10 centers	2016–2018 [*n* = 60] 2 centers	2019–2020 [*n* = 351] 5 centers	2021–2023 [*n* = 334 11 centers
Use of robot						
Robot thorax + robot abdomen	16 (34)	133 (75)	348 (54)	35 (58)	296 (84)	277 (83)
Robot thorax + laparoscopy	30 (64)	43 (24)	249 (39)	23 (38)	49 (14)	47 (14)
Robot thorax + laparotomy	1 (2)	0 (0)	2 (0)	2 (3)	6 (2)	8 (2)
Robot abdomen + thoracoscopy	0 (0)	0 (0)	6 (1)	0 (0)	0 (0)	1 (0)
Robot abdomen + thoracotomy	0 (0)	1 (1)	35 (6)	0 (0)	0 (0)	1 (0)
Patient positioning, thoracic phase						
Semi-prone	47 (100)	176 (99)	545 (85)	24 (40)	60 (17)	79 (24)
Left lateral decubitus	0 (0)	1 (1)	94 (15)	36 (60)	291 (83)	220 (66)
Prone	0 (0)	0 (0)	1 (0)	0 (0)	0 (0)	30 (9)
Other	0 (0)	0 (0)	0 (0)	0 (0)	0 (0)	5 (2)
Lymphadenectomy						
Mediastinal lymphadenectomy						
High – paratracheal nodes	37 (80)	117 (67)	248 (39)	38 (63)	149 (43)	162 (49)
Mid – subcarinal nodes	47 (100)	127 (72)	482 (75)	58 (97)	324 (92)	308 (92)
Low – para-esophageal nodes	47 (100)	153 (86)	614 (96)	45 (75)	275 (78)	316 (95)
Total lymph node yield (median [IQR])	30 [23–40]	35 [27–44]	33 [24–41]	26 [19–32]	26 [20–33]	30 [22–43]
Anastomosis						
Anastomotic technique						
Circular-stapled	1 (2)	123 (70)	377 (59)	30 (50)	278 (79)	230 (69)
Hand-sewn	46 (98)	48 (27)	96 (15)	24 (40)	31 (9)	55 (17)
Linear-stapled	0 (0)	6 (3)	167 (26)	1 (2)	42 (12)	48 (14)
No anastomosis	0 (0)	0 (0)	0 (0)	5 (8)	0 (0)	1 (0)
Anastomotic type						
End-to-side	37 (79)	171 (97)	450 (70)	54 (98)	306 (87)	269 (81)
End-to-end	9 (19)	0 (0)	15 (2)	0 (0)	2 (1)	46 (14)
Side-to-side	1 (2)	6 (3)	175 (27)	1 (2)	43 (12)	18 (5)
Postoperative outcomes						
Textbook outcome^a^	20 (43)	95 (54)	327 (51)	28 (47)	223 (64)	213 (64)
Operative time, min (median [IQR])	384 [349–428]	389 [338–435]	405 [354–461]	271 [229–352]	282 [238–345]	317 [263–428]
Postoperative complications						
Any	38 (81)	102 (58)	365 (57)	26 (43)	175 (50)	173 (52)
Pulmonary	20 (43)	50 (28)	157 (25)	20 (33)	51 (15)	62 (19)
Pneumonia	12 (26)	35 (20)	124 (19)	9 (15)	26 (7)	31 (9)
Anastomotic leakage	13 (28)	31 (18)	98 (15)	8 (13)	40 (11)	34 (10)
Recurrent laryngeal nerve injury	1 (2)	9 (5)	13 (2)	3 (5)	93 (27)	79 (24)
R0 resection^b^	44 (94)	170 (96)	598 (95)	48 (80)	304 (87)	299 (91)
Length of stay						
ICU/MCU stay, days (median [IQR])	2 [1–3]	1 [1, 2]	2 [1–4]	2 [0–3]	1 [1, 2]	1 [1–3]
Hospital stay, days (median [IQR])	11 [8–17]	12 [11–19]	12 [9–19]	10 [8–11]	9 [8–12]	9 [8–14]
Mortality <30 days	1 (2)	6 (3)	15 (2)	3 (5)	4 (1)	6 (2)

Data are expressed as *n* (%) unless otherwise specified

Percentages may not equal 100% due to rounding

*UGIRA* Upper GI International Robotic Association, *IQR* interquartile range, *ICU* intensive care unit, *MCU* medium care unit

^a^Definition: complete resection (R0), no intraoperative complications, a lymph node yield of ≥ 15, no Clavien–Dindo ≥ 3 complications, no readmission to the ICU/MCU, no re-interventions, no hospital readmission after discharge, no length of hospital stay > 21 days, no mortality < 30 days, and no in-hospital mortality [16]

^b^Refers to R0 as defined by the College of American Pathologists (i.e. absence of malignant cells within the resection margins)

To create the esophagogastric anastomosis in Ivor–Lewis and McKeown procedures, circular stapling was used in 2%, 70%, and 59%, and 50%, 79%, and 69%; linear stapling was used in 0%, 3%, and 26%, and 2%, 12%, and 14%; and a handsewn technique was used in 98%, 27%, and 15%, and 40%, 9%, and 17%, respectively. Median operating time was 384, 389, and 405 min for Ivor–Lewis procedures, and 271, 282, and 317 min for McKeown procedures.

Postoperative complications occurred after 81%, 58%, and 57% of Ivor–Lewis procedures, and 43%, 50%, and 52% of McKeown procedures (Fig. 2b). Anastomotic leakage rates were 28%, 18%, and 15% in Ivor–Lewis cases, and 13%, 11%, and 10% in McKeown cases (Fig. 2b). R0 resection rate was 94%, 96%, and 95% for Ivor–Lewis cases, and 80%, 87%, and 91% for McKeown cases (Fig. 2d). In the Ivor–Lewis and McKeown groups, hospital stay was 11, 12, and 12 days, and 10, 9, and 9 days, respectively (Fig. 2e).

## Discussion

This study provides an overview of the current state of RAMIE, and the progression of its operative techniques and perioperative outcomes within an extensive international cohort over recent years. The results show a favorable tendency to achieving textbook outcome after RAMIE and demonstrate outcomes comparable with previous benchmarks.^18,19^ Additionally, these findings facilitate the identification of areas for improvement.

Our primary outcome is a composite measure that describes an optimal perioperative course, and is associated with better long-term survival.^16,20^ In combination with our data being derived from a registry, that introduces the possibility of missing data, the stringent criteria for achieving textbook outcome (i.e., Clavien–Dindo score, lymph node yield, length of stay), can even result in an underestimation of our primary outcome.

Contrary to expectations, our results did not show more fully robotically performed procedures over time. During the introduction of RAMIE, it is common for most centers to initially perform the procedure as a hybrid, involving both robotic assistance and other surgical approaches, such as laparoscopy, thoracoscopy, laparotomy or thoracotomy.^21^ Particularly in the 2016–2018 time group, center bias from the first involved UGIRA centers may have influenced the results. However, results from the sensitivity analysis did not show more fully robotic RAMIE procedures over time in experienced centers. Importantly, there was a substantial number of cases involving an open thoracic phase in this study, even in centers experienced regarding the robotic abdominal phase. While the number of hybrid procedures can be partly explained by the learning curve, it also reflects the gradual transition from minimally invasive to robot-assisted techniques in such complex, multi-phase procedures. Despite some studies suggesting that RAMIE may be associated with less postoperative complications compared with MIE, these real-world results highlight the ongoing need for robust evidence to ascertain which approach offers optimal outcomes with minimal morbidity.^11,12^ Our findings underscore this necessity and advocate for a continued emphasis on the adoption of minimally invasive techniques as an area for improvement.

Lymph node yield has been established as a significant prognostic factor for long-term survival after esophagectomy.^22^ A meta-analysis has demonstrated that patients undergoing RAMIE have a greater number of harvested lymph nodes compared with those undergoing MIE.^23^ Our results indicate a relatively high median lymph node retrieval for both Ivor–Lewis and McKeown procedures over time (27, 30, and 30, and 26, 26, and 34 nodes, respectively), exceeding the benchmarks set in 2017 for conventional MIE and mean lymph node yields of the recent meta-analysis comparing MIE and RAMIE (22 in MIE, 23 in RAMIE).^18,23^ These observations are consistent in experienced RAMIE centers, where comparable numbers were observed (30, 35, and 33 nodes in Ivor–Lewis procedures, and 26, 26, and 30 nodes in McKeown procedures). Notably, despite the consistent relatively high lymph node yield, the performance of high mediastinal lymphadenectomy appears to decline over time, which is also influenced by center bias of the first UGIRA centers that routinely conducted a high mediastinal lymphadenectomy.

Even though oncological results are not consistently reported in the literature, R0 resection rates range between 81 and 100%.^24^ In our cohort, the R0 resection rate for Ivor–Lewis cases is 94% and higher, whereas in the McKeown group, the percentages appear notably lower (≤ 92%). Among patients within the McKeown group, 42% did not undergo neoadjuvant therapy before surgery, with a predominant representation from a single Asian center, potentially impacting the R0 resection rates.^25,26^

In terms of postoperative complications, the current results seem to be comparable with previous benchmark studies.^18,19^ Despite the diversity in anastomotic techniques, the results demonstrate a declining rate of anastomotic leakage over time for both Ivor–Lewis (22%, 22%, 16%) and McKeown (14%, 12%, 11%) procedures. The anastomotic leakage rates within our experienced cohorts (28%, 18%, and 15% for Ivor–Lewis; 13%, 11%, and 10% for McKeown) indicate the potential for future improvement in both Ivor–Lewis and McKeown procedures. At the same time, these rates reflect the high surgical quality achieved in the current cohort compared with the benchmark value for anastomotic leakage (≤ 20%) of conventional MIE.^18^ Additionally, further research is needed to investigate potential associations between patterns of practice and anastomotic leakage rates.

Compared with a recent meta-analysis showing a 15-day hospital stay after RAMIE, our findings reveal shorter median hospital stay in both Ivor–Lewis (13, 14, 13 days) and McKeown (12, 9, 9 days) procedures.^23^ For the experienced centers, the results demonstrate comparable outcomes with fewer days, especially in the Ivor–Lewis group (11, 12, 12 days), indicating that there is still potential for further improvement. Implementation of enhanced recovery after surgery (ERAS) protocols, providing a format for multidisciplinary care, contribute to shortening of hospital stay.^27,28^

This study stands as the largest RAMIE cohort to date, with participation from 28 centers across four continents, providing a comprehensive and representative overview of current global RAMIE practices. Moreover, it is the first study to present worldwide outcomes over time for RAMIE procedures and to employ these to address areas for future improvement.

Nonetheless, this study has limitations that warrant consideration. First, it is essential to acknowledge that this is a descriptive study from a worldwide heterogeneous cohort and with various new centers joining during the inclusion period. This limits our ability to draw definitive conclusions. Second, centers were at different stages of their RAMIE learning curve upon commencing data registration in the UGIRA Esophageal Registry. While the sensitivity analysis was utilized to mitigate such bias, determining the cut-off for the learning curve remains debatable as its exact duration varies largely depending on studied outcome, surgeon experience, and technique.^13^ Third, it is important to mention the potential influence of center bias, particularly in the initial groups (2016–2018). Lastly, tumor localization, which might also influence R0 resection rates, is not captured in the registry.

## Conclusion

This study demonstrates favorable outcomes over time regarding achievement of textbook outcome after RAMIE, reflecting the safe implementation of RAMIE over recent years. Areas for improvement include further reduction of anastomotic leakage rates and shortening of length of hospital stay. Moreover, areas for further investigation include the evaluation of a fully robotic technique. The UGIRA initiative may contribute to optimizing the RAMIE technique by providing insight into its global status over time.

## Supplementary Information

Below is the link to the electronic supplementary material.

Supplementary file1 (DOCX 23 kb)
