# Supplementary material for: The Current State of Robot-Assisted Minimally Invasive Esophagectomy (RAMIE): Outcomes from the Upper GI International Robotic Association (UGIRA) Esophageal Registry
Source: Ann Surg Oncol. 2024 Nov 4;32(2):823–33. doi: 10.1245/s10434-024-16364-9 (PMC11698755; doi:10.1245/s10434-024-16364-9)
Supplement: Supplementary file 1 — Supplementary file1 (DOCX 23 kb) [file 10434_2024_16364_MOESM1_ESM.docx]

**Supplementary Table 1:** The number of centers that registered Ivor-Lewis esophagectomy cases in the Upper GI International Robotic Association (UGIRA) Esophageal Registry per continent, stratified per time period.

|  | **Ivor-Lewis cases** | | |
| --- | --- | --- | --- |
| **Primary analysis** | ***2016-2018***  ***(n=368)***  ***12 centers*** | ***2019-2020***  ***(n=563)***  ***16 centers*** | ***2021-2023***  ***(n=1081)***  ***20 centers*** |
| **Continent**  Europe  North America  Asia  South America | 10 centers. n=295  1 center, n=31  1 center, n=42  0 centers, n=1 | 14 centers, n=536  1 center, n=23  1 center, n=4  0 centers, n=0 | 18 centers, n=1041  1 center, n=34  1 center, n=6  0 centers, n=0 |
| **Sensitivity analysis** | ***2016-2018***  ***(n=47)***  ***3 centers*** | ***2019-2020***  ***(n=177)***  ***5 centers*** | ***2021-2023***  ***(n=640)***  ***10 centers*** |
| **Continent**  Europe  North America  Asia  South America | 3 centers, n=47  0 centers, n=0  0 centers, n=0  0 centers, n=0 | 5 centers, n=177  0 centers, n=0  0 centers, n=0  0 centers, n=0 | 9 centers, n=619  1 center, n=21  0 centers, n=0  0 centers, n=0 |

**Supplementary Table 2:** The number of centers that registered McKeown esophagectomy cases in the Upper GI International Robotic Association (UGIRA) Esophageal Registry per continent, stratified per time period.

|  | **McKeown cases** | | |
| --- | --- | --- | --- |
| **Primary analysis** | ***2016-2018***  ***(n=275)***  ***11 centers*** | ***2019-2020***  ***(n=406)***  ***15 centers*** | ***2021-2023***  ***(n=499)***  ***17 centers*** |
| **Continent**  Europe  North America  Asia  South America | 5 centers, n=69  1 center, n=2  4 centers, n=182  1 center, n=22 | 9 centers, n=47  1 center, n=1  4 centers, n=356  1 center, n=2 | 12 centers, n=129  0 centers, n=0  5 centers, n=370  0 centers, n=0 |
| **Sensitivity analysis** | ***2016-2018***  ***(n=60)***  ***2 centers*** | ***2019-2020***  ***(n=351)***  ***5 centers*** | ***2021-2023***  ***(n=334)***  ***11 centers*** |
| **Continent**  Europe  North America  Asia  South America | 1 center, n=24  0 centers, n=0  1 center, n=36  0 centers, n=0 | 3 centers, n=31  0 centers, n=0  2 centers, n=320  0 centers, n=0 | 8 centers, n=75  0 centers, n=0  3 centers, n=259  0 centers, n=0 |

**Supplementary Table 3:** Baseline characteristics of experienced centers (from case 71 and onwards) including patients in the Upper GI International Robotic Association (UGIRA) Esophageal Registry (n=1609)

|  | **Ivor-Lewis (n=864)**  ***11 centers*** | **McKeown (n=745)**  ***12 centers*** |
| --- | --- | --- |
| **Continent**  Europe  North America  Asia  South America | 843 (98)  21 (2)  0 (0)  0 (0) | 130 (17)  0 (0)  615 (83)  0 (0) |
| **Age,** *years (median [IQR])* | 66 [59 – 72] | 66 [60 – 71] |
| **Sex**  Male  Female | 705 (82)  159 (18) | 602 (81)  143 (19) |
| **BMI,** *kg/m^2^ (mean [SD])* | 26.1 [± 4.5] | 23.4 [± 3.6] |
| **ASA-classification**  1  2  3  4 | 65 (8)  459 (54)  321 (38)  8 (1) | 14 (2)  456 (61)  264 (35)  6 (1) |
| **Any comorbidity**  Pulmonary comorbidity  Cardial comorbidity  Vascular comorbidity  Oncological comorbidity  Neurological comorbidity  Diabetes | 632 (73)  127 (15)  276 (32)  261 (30)  70 (8)  54 (6)  118 (14) | 360 (48)  27 (4)  53 (7)  231 (31)  24 (3)  11 (2)  84 (11) |
| **Histology**  Adenocarcinoma  Squamous cell carcinoma | 731 (85)  133 (15) | 88 (12)  657 (88) |
| **Clinical T-stage**  cT1a  cT1b  cT2  cT3  cT4a  cT4b  Missing | 11 (1)  58 (7)  164 (19)  600 (69)  23 (3)  5 (1)  3 (0) | 11 (2)  27 (4)  246 (33)  422 (57)  12 (2)  24 (3)  3 (0) |
| **Clinical N-stage**  cN0  cN+ (cN1 – cN3)  Missing | 308 (36)  555 (64)  1 (0) | 192 (26)  549 (74)  4 (1) |
| **Neoadjuvant therapy**  None  Chemotherapy  Radiotherapy  Chemoradiotherapy  Other  Missing | 98 (11)  223 (26)  2 (0)  531 (62)  6 (1)  4 (1) | 356 (48)  133 (18)  4 (1)  230 (31)  22 (3)  0 (0) |
| **Robotic system**  Da Vinci S  Da Vinci Si  Da Vinci X  Da Vinci Xi | 0 (0)  22 (3)  14 (2)  828 (96) | 161 (22)  253 (34)  3 (0)  328 (44) |

Abbreviations: BMI = Body Mass Index (kg/m^2^); ASA = American Society of Anesthesiologists. SD = standard deviation; IQR = Interquartile range.

Percentages may differ from 100% due to rounding.
